# Minimal Invasive Pre-Op CT-Guided Gold-Fiducials in Local Anesthesia for Easy Level Localization in Thoracic Spine Surgery

**DOI:** 10.3390/jcm13195690

**Published:** 2024-09-25

**Authors:** Fee Keil, Frank Hagemes, Matthias Setzer, Bedjan Behmanesh, Gerhard Marquardt, Elke Hattingen, Vincent Prinz, Marcus Czabanka, Markus Bruder

**Affiliations:** 1 Institute of Neuroradiology, University hospital Frankfurt, 60528 Frankfurt am Main, Germany; elke.hattingen@unimedizin-ffm.de; 2 Department of Neurosurgery, University hospital Frankfurt, 60528 Frankfurt am Main, Germanymatthias.setzer@unimedizin-ffm.de (M.S.); bedjan.behmanesh@med.uni-rostock.de (B.B.); gerhard.marquardt@unimedizin-ffm.de (G.M.); vincent.prinz@unimedizin-ffm.de (V.P.); marcus.czabanka@unimedizin-ffm.de (M.C.); 3 Department of Neurosurgery, Kantonspital Aarau, 5001 Aarau, Switzerland; markus.bruder@ksa.ch

**Keywords:** intraoperative level localization, minimal invasive, CT-guided fiducial placement, local anesthesia markers, preoperative spinal procedures

## Abstract

**Background:** The accurate identification of intraoperative levels is of paramount importance in spinal surgery, particularly in cases of obesity or anatomical anomalies affecting the thoracic spine. The aim of this work was to clarify whether the preoperative percutaneous placement of fiducial markers under local anesthesia only, with minimal discomfort to the patient, can be performed safely and efficiently. **Methods:** Patients treated at our institution between June 2019 and June 2020 for thoracic intraspinal lesions with preoperative percutaneous gold fiducial placement were analyzed. A total of 10 patients underwent CT-guided gold fiducial placement 2–48 h prior to surgery on an outpatient or inpatient basis. Patient characteristics, CT intervention time, and perioperative complications were recorded. **Results:** In all cases, the gold markers were placed under local anesthesia alone and were easily visualized intraoperatively with fluoroscopy. There was no preoperative dislocation or malposition. The procedure was performed without X-ray exposure to the neuroradiology interventionalist. The average CT intervention time from the planning scout to the final control time was 14.3 min. The percentage of anatomical norm variants in our observation group was high, as 2 of the 10 patients had lumbarization of the first sacral vertebra, resulting in a six-link lumbar spine. **Conclusions:** Preoperative CT-guided transcutaneous submuscular placement of gold markers under local anesthesia is a practical and safe method for rapid and accurate intraoperative level determination in thoracic spine surgery in a time-saving minimally invasive manner. The virtually painless procedure can be performed either preoperatively on an outpatient basis or as an inpatient procedure.

## 1. Introduction

Intraoperative level determination is a critical step in spine surgery, but can be challenging, especially in the thoracic spine, and remains a topical issue [1]. 

In an anonymized survey by Meyer et al., 2014 [2], 68% of all spine surgeons reported operating at the wrong height at least once during their career.

The difficulty is often due to factors such as obesity, which makes it difficult to visualize anatomical structures during intraoperative fluoroscopy, anatomical variations such as transitional vertebrae that are difficult to image, or a number of vertebral bodies or ribs that deviate from the norm [3,4,5]. 

Operating at the wrong level can lead to significant complications that affect both patient safety and surgical outcomes. Despite advances in medical imaging and navigation techniques, this problem remains relevant because there are no uniform standards for preoperative marking in thoracic spine surgery [6]. 

In other surgical specialties, the concept of preoperative pathology marking has proven successful when intraoperative identification of pathology is difficult. In breast surgery, for example, wire or seed marking is often used to locate small or nonpalpable lesions preoperatively [7]. In stereotactic radiosurgery, gold markers are placed in tumors to allow precise targeting during treatment [8], and in spinal fistulas or spinal arteriovenous malformations, endovascular coils can be used to preoperatively mark important vascular structures prior to neurosurgical procedures to facilitate intraoperative navigation [9]. 

Each of these procedures uses specific markers and techniques tailored to the anatomic and pathologic conditions.

There is a lack of standards for preoperative or intraoperative level markers in thoracic spine surgery, although studies have shown that preoperative level markers can be of great benefit in spine surgery, particularly in the case of thoracic spine surgery [6]. These markers can increase the accuracy of surgery and reduce the risk of complications, but at present, there is no agreed technique. 

The various spine-level marking techniques described in the literature to date range from only intraoperative navigation for thoracic spine procedures [10] and techniques where precise localization of the target height is achieved by preoperative CT scans combined with intraoperative fluoroscopy to reduce the X-ray radiation for the surgical team [11,12,13]. 

Methods associated with less X-ray exposure to personnel, such as transcutaneous wire marking performed immediately prior to surgery or intraosseous marking, can also be performed with fluoroscopy and additional computed tomography (CT). But all the techniques described so far required at least conscious sedation, if not general anesthesia [6].

The risk of surgery at the wrong level increases in the absence of preoperative marking, particularly in the presence of anatomical norm variants such as numerical deviations or the presence of assimilation vertebrae, which make classification more difficult [14], as the intraoperative counting method is often from L5 to cranial. Overall, norm variants such as transitional vertebrae are not uncommon. Transitional vertebrae are estimated to have a prevalence of approximately 12.6% in the normal population [15], and numerical deviations are even more common [4].

While these methods are promising, there are challenges. The need for anesthesia, whether general anesthesia or sedation with monitoring of vital signs, can be a significant barrier to clinical procedures and complicates implementation in both the prehospital and outpatient settings due to the interdisciplinary coordination required. 

In the inpatient setting, immediate preoperative marking requires good interdisciplinary coordination between interventional radiology, anesthesia, and the surgeon. The involvement of multiple time-sensitive disciplines not only increases the complexity and cost of the procedure but also poses additional risks to the patient. The use of intraoperative navigation requires specialized equipment and experienced personnel, limiting the availability and widespread use of these methods.

To address these challenges, we have developed a new minimally invasive technique that allows CT-guided percutaneous marking under local anesthesia alone. This method has the potential to make preoperative level marking safer and more accessible by eliminating the need for general anesthesia while ensuring high accuracy. We analyzed the method regarding feasibility and patient safety. 

## 2. Materials and Methods

Having been approved by the institutional ethical review board, we retrospectively analyzed the data of all patients who underwent percutaneous fiducial gold marker placement before thoracic spine surgery at our institution from June 2019 to June 2020. Prior to the procedure, informed consent for the fiducial gold marker placement of every single patient was taken. The principles of the Helsinki Declaration of 1964 and its later amendments were followed. Patient data were collected by reviewing surgery reports, radiological findings, image documentation, and physician letters. In addition to patient characteristics such as gender, age, underlying surgical indication, and anatomical standard variants of the spine, the localization of the spinal pathology and the date of marking with localization of the marking were evaluated. Furthermore, the CT-intervention time, intraoperative fluoroscopy time, and complications such as postoperative bleeding, infection, or occurrence of a dural leak were recorded as well.

### 2.1. Gold Marker Placement

CT-controlled gold marking was performed by an interventional neuroradiologist on the day of surgery or a maximum of 72 h before the planned neurosurgery, using a computer tomograph (Philips Ingenuity 128 core, Amsterdam, The Netherlands). Considering the previous CT and MRI examinations, a CT planning scout was created. The procedure was performed partly on an inpatient basis, but in two of the ten patients, it was also performed on an outpatient basis in the pre-inpatient setting the day before admission for surgery. 

The patient was positioned as comfortably as possible in the prone position on the CT table. The patient’s arms were either placed under the patient’s head, which was turned to the side or alternatively, stretched out to either side of the head.

Initially, a counting scout was prepared. The target vertebral body was determined by taking into account all of the available previous images (CT and/or MRI images).

**Figure 1:**
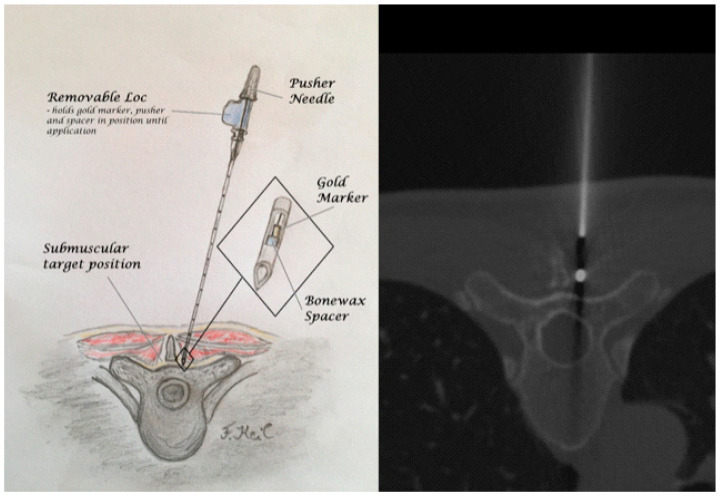
Gold marker application: CT-scan with an application needle and illustration of the target area with a preloaded needle (Mick^®^ Fiduciary) and gold marker.

A thin-layer CT planning spiral with a grid positioned on the skin was then produced over the target region only. After exact target localization in the axial CT images, the target layer was determined, and the laser was adjusted at the planning level. This was followed by skin marking using a pen or pressure marker and extensive skin disinfection. Under sterile conditions, local anesthesia with 5 mL Mepivacaine 5% with a small stab incision was applied. Subsequently, laser-orientated insertion of the 1 × 3 mm measuring gold marker (Mick^®^ fiduciary gold marker, 5203 Bristol, GA 30518, USA) was performed via a 20 cm 18G preloaded introducer needle with a pre-waxed tip, aiming at the pedicle of the target vertebral body. The marker was placed in the periosteal–submuscular layer illustrated in Figure 1. CT single-slice images were used to verify the correct position of the delivery needle and gold marker. The interventionalist left the CT room for the control images and was not exposed to radiation at any time.

**Figure 2:**
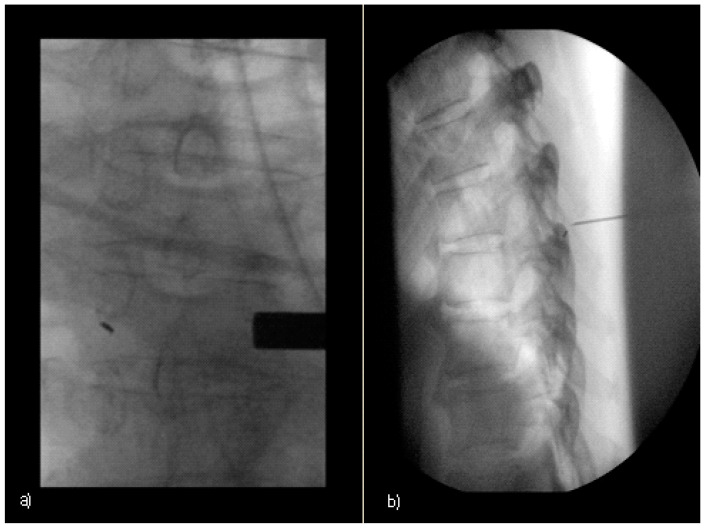
Intraoperative gold marker identification with fluoroscopy: (**a**) 68-year-old patient (Table 1, patient number 10) anterior–posterior view (**b**) 56-year-old patient (Table 1, patient number 6) additional lateral view with a additional guiding needle.

After the removal of the needle, a plaster bandage was applied, and a final short CT spiral was performed only over the area of the applied marker to verify the exact marker position. Positioning the marker contralaterally to the planned surgical access route protects against accidental intraoperative dislocation or extirpation. Intraoperatively, the target level was identified via fluoroscopic identification of the preoperative-placed gold marker, as shown in Figure 2. 

### 2.2. Intraoperative Level Localization

In the operating room (OR), the patient was placed in a stable prone position under general anesthesia. The surgical area was roughly defined using standard externally palpable anatomical landmarks, with the 1–2 mm skin incision made a maximum of 48 h prior to gold marking still visible in all patients. After extensive skin disinfection and sterile draping, the exact target level was verified by a single-shot C-arm image. Figure 2a shows the identification of the clearly visible preoperative gold marker.

A needle was then inserted percutaneously close to the contralateral lamina of the marked vertebral body. The needle serves as a guide and enables reliable preparation of the target plane. In case of uncertainties, the correspondence between the localization of the gold marker localization and the needle tip placement could be verified by means of an additional lateral image (Figure 2b).

Gold marker localization was generally performed using only one single shot image in the anterior–posterior orientation and additional lateral orientation as required by the operating surgeon.

### 2.3. Statistical Analysis

The means and standard deviation have been calculated. The results are presented as percentages along with the corresponding proportions. The tables were created in Excel version 22.

## 3. Results

During the observation period, 10 patients received preoperative gold marking prior to spinal surgery according to the standard procedure of the Institute for Neuroradiology of our clinic. Patient characteristics are shown in Table 1. 

All preoperative gold markings could be performed under local anesthesia without conscious sedation during the procedure. In eight patients, the initial imaging covered the whole spine from the craniocervical junction to the os sacrum; in two patients, the available imaging covered only the thoracic spine. Consistent with the frequently occurring deviation in the number of vertebral bodies described in the literature before [3,4,16], two of the eight patients with full spine imaging showed a transition anomaly. Both patients had a lumbarization of the first sacral vertebra with a consecutive six-link lumbar spine. 

Presurgical gold-marking was indicated by the responsible neurosurgeon. The preoperative diagnoses were intraspinal meningiomas (30%), schwannomas (30%), a malignant peripheral nerve sheath tumor (10%), spinal CSF leakage (10%), a herniated disc (10%), and spinal cord herniation (10%). The spinal level of the pathology, preoperative indications, and the level of the surgical intervention are shown in Table 1; the time of neuroradiological intervention and the intraoperative fluoroscopy time are shown in Table 2.

The average CT-intervention time between the planning scout and the final CT control scan was 14.3 min. In all cases, the marker could be detected easily during surgery by fluoroscopy without the necessity of fluoroscopic visualization of additional anatomical landmarks (Figure 2). 

There was no case of preoperative dislocation or misplacement. Intraoperative localization of the gold marker corresponded to the level of the pathology in all cases.

No gold-marking-related or intraoperative complication occurred in any of the 10 observed patients. Two patients underwent revision surgery, one because of a subcutaneous cerebrospinal fluid collection and another because of postoperative hemorrhage due to previously unknown platelet dysfunction. 

The reoperation was not related to the gold marking. Both patients were discharged for rehabilitation in good condition.

## 4. Discussion

CT-controlled marking of the surgical level by means of preoperative gold markings can significantly facilitate intraoperative level localization [13], especially in the region of the thoracic spine. Particularly in patients with obesity or unconventional spinal anatomy, previous studies have shown that preoperative level marking reduces the risk of wrong-level surgery [9,11,12]. 

The use of traditional intraoperative fluoroscopic localization for surgeries involving the middle and upper thoracic spine is often influenced by individual patient anatomy, such as body mass index (BMI), spinal curvature (kyphosis), and the presence of transitional vertebrae. These anatomical variations can complicate the accuracy and speed of fluoroscopic techniques, leading to longer procedures and requiring repeated different angled X-rays. This not only increases the duration of the surgery but also exposes both the patient and the surgical team to additional radiation. In contrast, the use of a gold marker provides a precise and efficient method for intraoperative spinal-level localization. By placing the marker preoperatively, surgeons can significantly reduce the number of intraoperative X-ray images needed. Typically, the information required can be obtained from a single AP X-ray, which may be supplemented by a lateral image with guiding needle placement if necessary. This reduction in the number of X-rays is crucial for minimizing radiation exposure even for the operation team and can shorten the duration of surgery. 

It would be ideal if the gold marker application could be integrated into the standard preoperative CT examination, as this would save patients from having to undergo an additional CT examination altogether. However, the majority of patients come to the operating hospital with CT or MRI scans that have already been performed externally. Furthermore, a CT scan is not always required after an MRI scan, or the decision to operate is only made after the imaging has been carried out. Despite good planning and interdisciplinary coordination, an additional CT scan for gold marker placement can only be avoided in a small proportion of patients. Nevertheless, the benefits of preoperative marker placement are considerable: it eliminates the need for time-consuming intraoperative fluoroscopic counting of vertebrae, which can be particularly challenging in patients with complex anatomies. Once the marker is placed, its location relative to the vertebrae on the intraoperative image in the anterior–posterior orientation can be directly compared to the preoperative CT images with the marker, ruling out dislocation and ensuring accurate localization. The gold marker is placed subcutaneously near the vertebral lamina, a location that is highly stable and less prone to movement. Moreover, the short interval between marker placement and surgery—typically within 48 h, but after a maximum of 72 h—further reduces the likelihood of dislocation. In our experience and in this admittedly small cohort study, no marker movement has been observed during this window. However, further studies with larger patient populations are necessary to confirm these findings and establish long-term reliability.

Ultimately, the minimal risks associated with the additional CT procedure that may be necessary for gold marking, including the cost of materials, are outweighed by the significant advantages of increased surgical safety, enhanced precision, reduced intraoperative radiation exposure, and a potential reduction in operating time.

The data of our pilot study clearly demonstrate the feasibility and safety of the submuscular placement of the marker on the lamina of the corresponding vertebral body. For this approach, neither an anesthetist nor special monitoring of the vital parameters is necessary while the patient is awake for the marker positioning procedure. The procedure was tolerated well by all patients without sedation.

The implantation of other previously used markers, such as intraosseous markers, however, is only feasible under general anesthesia or at least analgesic sedation due to the pain [8]. 

Due to the small diameter of the introducer cannula, neither the application of skin glue nor a skin suture was necessary. As the gold marker is released directly submuscular, the patient can move freely afterward without fear of accidental dislocation, in contrast to transcutaneous marking wires used in the past [17]. In contrast to markings placed under fluoroscopy guidance, the position of the marker is checked immediately when the procedure is performed under CT control [8]. Unnecessary repositioning of the patient, further logistical problems due to transport, or time-consuming scheduling between staff of different units or other departments could be avoided. The gold marker could be reliably visualized intraoperatively at the predicted level in all patients and led the surgeons directly to the target pathology. In none of the cases was wrong level access performed. The previously described use of gold markers was intraosseous [18,19,20]. Displacement with submuscular placement is theoretically conceivable but was not observed in our feasibility study with implantation up to 72 h prior to surgery.

The limitations of this study are that it provides only preliminary results and that the number of cases is small. This is a descriptive study to analyze the feasibility of the method and patient safety.

However, the excellent performance of the procedure should encourage us to perform a prospective cross-sectional study comparing a patient cohort prepared with this new gold-fiducial placement under CT guidance with an adequate control group. 

## 5. Conclusions

Preoperative CT-controlled transcutaneous submuscular gold marker placement using local anesthesia enables quick and precise intraoperative level identification in thoracic spine surgery in a time-spearing and minimally invasive manner. The presented technique is feasible and safe and can be performed in both outpatient and inpatient settings prior to surgery. Further examinations should show if the presented procedure has a relevant impact on operating time or intraoperative radiation exposure. 

## Figures and Tables

**Table 1:** Patient’s data n = 10.

Nr.	Gender	Age	Marker Localization	Level of Pathology	Surgery Level	First Sacral Vertebra Lumbarization	Diagnosis
1	female	82	Th 9	8–9	8–9	yes	Schwannoma
2	male	74	Th 8	8–9	8–9	no	Schwannoma
3	female	65	Th 4	3–5	4	no	Meningioma
4	female	82	Th 6	6–7	6–7	no	Meningioma
5	female	57	Th 2	1–2	1–2	no	CSF leakage
6	female	56	Th 8	8–9	7–9	no	Meningioma
7	male	48	Th 6	6–7	6–7	no	Disc herniation
8	male	32	Th 3	3–4	3–4	no	Schwannoma
9	female	57	Th 7	6–7	6–7	yes	Cord herniation
10	male	68	Th 3	3–6	3–4	no	MPNST *

* MPNST = malignant peripheral nerve sheath tumor; Th = thoracic level; CSF = cerebrospinal fluid.

**Table 2:** Complications and time recording.

Nr.	Gold Marker Application without Complications	Interventional CT-Time (in Minutes)	Reliable Intraoperative Gold Marker Identification	Intraoperative Fluoroscopy Time (in Seconds)	Intra- Operative Complications	Wrong Level Surgery
1	yes	20	yes	0.1	no	no
2	yes	13	yes	0.06	no	no
3	yes	15	yes	0.09	no	no
4	yes	12	yes	0.2	no	no
5	yes	12	yes	0.09	no	no
6	yes	10	yes	0.07	no	no
7	yes	17	yes	0.06	no	no
8	yes	11	yes	0.2	no	no
9	yes	16	yes	0.19	no	no
10	yes	17	yes	0.35	no	no

## Data Availability

This study is a retrospective analysis, and as such, the data supporting the findings are not publicly available due to privacy and ethical restrictions. No new data were generated for this study, and the patient data used are protected to ensure confidentiality. Therefore, access to these data is restricted.
